# New Diseases Related to Cardiac Implantable Electronic Devices (CIEDs): An Overview

**DOI:** 10.3390/jcm14041322

**Published:** 2025-02-17

**Authors:** Pasquale Crea, Federica Cocuzza, Salvatore Bonanno, Nicola Ferrara, Lucio Teresi, Diego La Maestra, Paolo Bellocchi, Antonino Micari, Alice Moncada, Antonio Micari, Gianluca Di Bella, Giuseppe Dattilo

**Affiliations:** Cardiology Unit, Department of Clinical and Experimental Medicine, University of Messina, Via Consolare Valeria, 98124 Messina, Italy; federica.cocuzza93@gmail.com (F.C.); salvobonanno1@gmail.com (S.B.); nicolaferrara9650@gmail.com (N.F.); lucioteresi@gmail.com (L.T.); diego_lamaestra@unime.it (D.L.M.); paolo.bellocchi@yahoo.it (P.B.); antoniomicari96@gmail.com (A.M.); alycemoncada@gmail.com (A.M.); amicari@unime.it (A.M.); gianluca.dibella@unime.it (G.D.B.); gdattilo@unime.it (G.D.)

**Keywords:** CIED complications, pacemaker, implantable cardioverter defibrillator, resynchronization therapy

## Abstract

The widespread use of Cardiac Implantable Electronic Devices (CIEDs) has transformed the management of cardiac arrhythmias, improving survival and quality of life for millions. However, this progress has introduced a range of device-related complications, which can significantly impact patient outcomes. This review examines “new diseases” linked to CIEDs, categorizing them into physical (e.g., infections, venous obstruction, lead failure, and device recalls) and functional complications (e.g., arrhythmias, pacemaker syndrome, and left ventricular dysfunction). Prevention and management strategies are emphasized. Emerging technologies, such as leadless devices, quadripolar leads, and remote monitoring systems, hold promise in reducing risks and enhancing patient care. Future directions include integrating artificial intelligence for real-time monitoring, improving device durability, and exploring novel materials to minimize infections and mechanical failures. Understanding CIED-related complications is essential for healthcare providers to balance the benefits and risks of these life-saving technologies.

## 1. Introduction

The widespread adoption of Cardiac Implantable Electronic Devices (CIEDs) has undeniably revolutionized the management of cardiac rhythm disorders, offering life-saving therapeutic solutions to millions of patients worldwide. These devices, including pacemakers, implantable cardioverter-defibrillators (ICDs), and cardiac resynchronization therapy (CRT) devices, have significantly improved survival and quality of life for individuals suffering from bradyarrhythmias, tachyarrhythmias, and heart failure. However, the increased utilization of CIEDs has also led to the emergence of a novel spectrum of device-related pathologies that were non-existent in the pre-CIED era. As implantation rates continue to rise, understanding and addressing these complications have become a growing challenge for clinicians and healthcare systems alike.

**Figure 1:**
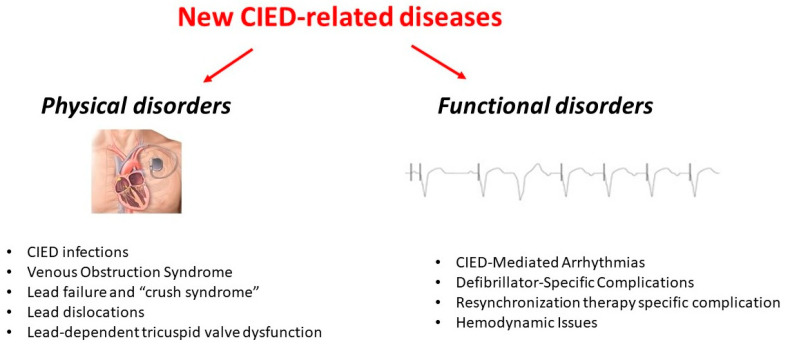
Overview of new complications associated with CIEDs.

CIED-related complications can be broadly categorized into physical and functional disorders (Figure 1). Physical complications encompass both infectious and structural device-related issues. Infectious complications, such as pocket infections and device-related endocarditis, remain a significant concern due to their potential for systemic spread and high morbidity. Structural device-related problems include lead fractures, which can result from mechanical stress or crush syndrome, cardiac perforation leading to pericardial effusion or tamponade, and manufacturing defects that may compromise the longevity and function of the device. Functional complications, on the other hand, manifest as disturbances in hemodynamics and arrhythmic events. Hemodynamic complications include left ventricular dysfunction due to chronic right ventricular pacing, a phenomenon increasingly recognized as a contributor to heart failure progression, and pacemaker syndrome, which results from the loss of atrioventricular synchrony. Arrhythmic complications encompass electrical storms, a life-threatening state of repetitive ventricular arrhythmias, and inappropriate shocks, which can significantly impact patient well-being and may even lead to psychological distress and reduced adherence to device therapy. Recognizing these “new diseases” associated with CIEDs is crucial for healthcare providers, as their prevention, early identification, and appropriate management play a pivotal role in optimizing patient outcomes. The complexity of these device-specific conditions highlights the necessity of a balanced and individualized approach in device therapy, weighing both the undeniable benefits and the potential risks associated with these technologies. The purpose of this review is to provide a comprehensive analysis of CIED-related complications by integrating both mechanical and functional issues into a unified framework. While previous reviews have often focused on either mechanical failures or functional disturbances in isolation, this work aims to bridge the gap by consolidating these aspects into the overarching concept of device-related disease. By doing so, we seek to enhance awareness and provide a more holistic understanding of the intricate challenges posed by CIED therapy, ultimately guiding better clinical decision making and improving patient care.

## 2. Physical Disorders

### 2.1. CIED Infections

**Figure 2:**
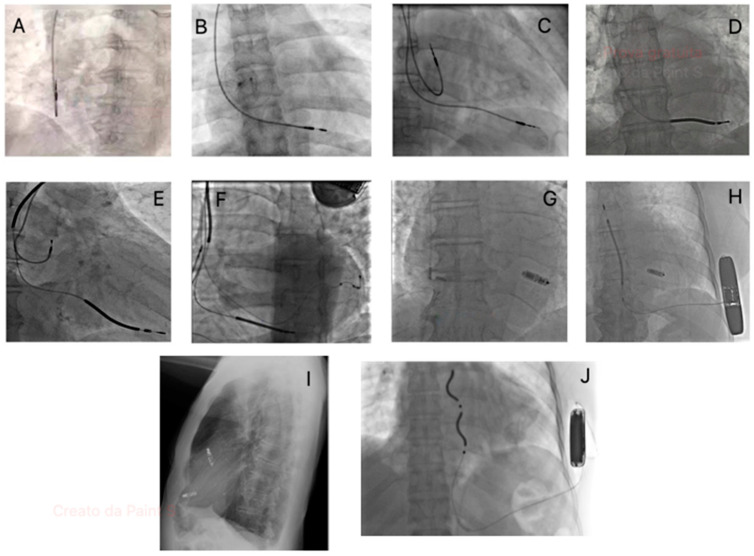
Fluoroscopic images showcasing different types of implantable cardiac devices: (**A**) Atrial single chamber pacemaker; (**B**) Ventricular single chamber pacemaker; (**C**) dual chamber pacemaker; (**D**) Single chamber ICD; (**E**) Dual chamber ICD; (**F**) CRT-D; (**G**) Leadless pacemaker (Micra-Medtronic, Minneapolis, MN, USA); (**H**) Leadless pacemaker (Micra-Medtronic, Minneapolis, MN, USA) plus subcutaneous ICD (Emblem-Boston Scientific, Marlborough, MA, USA); (**I**) Dual chamber leadless pacemaker (Aveir-Abbott, Abbott, Abbott Park, IL, USA); (**J**) Extravascular ICD (Aurora-Medtronic, Minneapolis, MN, USA).

Classic endovascular CIEDs implantations have spread significantly in recent decades (Figure 2).

This phenomenon is accompanied by a similar increase in CIED-related infections, which have an incidence of 1% to 4% of all CIED implantations [1], and a significant impact on mortality and morbidity, as well as on healthcare finances [2]. The increasing rate of these types of infection may be partially explained by the use of more complex devices in older patients [3]. The risk factors for CIEDs can be divided into patient-related and procedure-related factors. Regarding patient-related factors, there are non-modifiable factors such as age, male gender, chronic kidney disease, malignancy, chronic obstructive pulmonary disease, use of preprocedural temporary pacing, fever within 24 h before implantation, previous procedures and diabetes, and modifiable factors such as corticosteroid and anticoagulant or antiplatelet use [4]. The procedure-related risk factors include procedure duration, device revision or upgrade, device implant location in non-left pectoral, type of device (more leads and bigger device increase the risk), physician experience and the development of pocket hematoma [5,6]. Infections can occur in two ways: through contamination of the leads and/or generator, which can result in pocket infection and subsequent systemic infection, or through bloodstream infection from distant sites [2]. CIED infections can be classified into different nosographic entities. Superficial incisional infection is characterized by inflammation restricted to the skin and is the only case where a conservative approach, such as antibiotic therapy and close monitoring, may be sufficient. Incisional erosion occurs when the device perforates the skin in the absence of inflammation. Pocket infections involve the subcutaneous space housing the generator and can either be isolated or associated with bacteremia. Finally, CIED-related endocarditis may occur with or without a concurrent pocket infection [2,5]. Diagnosis is based on a combination of clinical, microbiological, and imaging findings. Physical examination is essential for detecting signs of pocket infection, including fluctuance, purulent drainage, device erosion through the skin, erythema, and fever [7]. Blood cultures should be performed before initiating antibiotic therapy, with at least three separate samples collected to identify the causative microorganism and to determine whether there is systemic involvement [8]. In cases of pocket infection, culturing the pocket material is also recommended. Imaging plays a key role, particularly transthoracic (TTE) and transesophageal echocardiography (TEE), which are used when there is high suspicion of systemic involvement, even in the presence of negative blood cultures. These modalities can detect lead or valvular endocarditic formations and are often essential for follow-up. Fluorine-18-fludeoxyglucose ([18F] FDG) positron emission tomography/computerized tomography (PET/CT) can be useful in differentiating superficial from systemic infections when the diagnosis of an isolated superficial pocket infection is uncertain [9]. Additionally, PET/CT may help identify embolic localizations of the infection. Currently, modified Duke criteria and ESC 2023 criteria are used for diagnosing infective endocarditis, although they are not specific for CIED infection. Coagulase-negative Staphylococci (CoNS) and Staphylococcus aureus are the most common bacteria isolated in CIED infections (60–90%). The remaining percentage is divided between gram-negative or other gram-positive bacteria (e.g., Pseudomonas aeruginosa or Enterococci) and, to a minimal extent, fungi (e.g., Candida). Many of these micro-organisms can create biofilm, an extracellular material that allows bacteria to attach to the device or lead and protect them from the antibiotic drugs and the host’s immune system activity. Electrophysiologists focus on preventing CIED infections through careful assessment of device indications and patient characteristics [10]. Prevention strategies for CIED infections include several key approaches. Antibiotic prophylaxis is recommended by current guidelines, which advise administering a second-generation cephalosporin one hour before the procedure, with vancomycin as an alternative [11]. However, the PADIT trial found no significant difference in infection reduction between pre-procedural cefazolin infusion alone and a reinforced perioperative antibiotic protocol [12]. Proper skin preparation is also essential. Chlorhexidine alcohol solution appears to be more effective than povidone-iodine in reducing infection incidence, though randomized data confirming this advantage is still lacking. Pocket hematoma assessment is another critical aspect of infection prevention, as pocket hematomas create a favorable environment for bacterial growth. Maintaining an accurate surgical technique with adequate hemostasis and proper wound closure is crucial. The BRUISE CONTROL 1 trial demonstrated that in patients receiving warfarin, discontinuing oral anticoagulants and using unfractionated heparin bridging therapy significantly increases the risk of pocket hematoma, with hematoma formation raising infection risk more than sevenfold [13]. The BRUISE CONTROL 2 trial, which investigated direct oral anticoagulant (DOAC) interruption in patients with atrial fibrillation and a CHA_2_DS_2_-VASc score of ≥2, found no significant differences in outcomes between treatment groups. Innovative tools such as antibacterial envelopes have also been explored [14]. The WRAP-IT trial demonstrated the efficacy of TyRX (Medtronic, Minneapolis, MN, USA), an absorbable antibacterial envelope that releases rifampicin and minocycline, in reducing infection rates among high-risk patients [15]. CIED-infection treatment is based on two pillars: device and leads extraction and antibiotic therapy. In consideration of the high in-hospital mortality rate (3–11%), complete CIED-extraction is critical, reducing mortality risk by approximately 39% compared to the antibiotic therapy alone [16]. Surgical removal is indicated for large endocarditis formation (>20 mm). Device extraction is not recommended in the case of single positive blood cultures without other clinical evidence of infection. The 2023 ESC guidelines on endocarditis [8] and the 2020 EHRA consensus on CIED infections [2] classify the duration of antibiotic therapy based on the type of infection. Surgical incision infections require 7 to 10 days of antibiotic treatment, while isolated pocket infections necessitate 10 to 14 days. In cases of systemic infection without vegetation on the valve or lead, post-extraction antibiotic therapy should be administered for 4 weeks, which can be reduced to 2 weeks if blood cultures are negative. Systemic infection with vegetation on valve or lead with or without embolism: 4 weeks post-extraction in case of native valve; from 4 to 6 weeks in case of prosthetic valve. In case of isolated pocket infection with prohibitive extraction risk (e.g., older age), an alternative approach called ultrahigh concentration of antibiotics (CITA) can be considered. This innovative protocol consists of a surgical procedure, in which is performed a surgical debridement with capsulectomy associated with pocket disinfection using alternating iodopovidone and hydrogen peroxide, and local antibiotic therapy via 6-F percutaneous catheter with loading dose followed by maintenance dose for 14 days in patients with positive cultures, otherwise for 10 days. Moreover, the wound was subjected to regulated negative pressure assisted therapy [17]. However, further randomized studies are needed to validate this approach. To avoid the prolongation of hospitalizations, the POET trial investigated the use of oral antibiotics in CIED-infection patients with stable conditions. The trial demonstrated the non-inferiority of oral antibiotic therapy compared to intravenous treatment, but more extensive studies are needed to confirm these findings [18]. After CIED-extraction, re-evaluation of re-implantation is necessary. Re-implantation should be delayed if there are signs or symptoms of infection or until blood cultures are negative for at least 3 days after extraction. The new device should be placed on the contralateral side. Recent advancements in cardiac implantable electronic devices (CIEDs) have introduced novel technologies aimed at reducing complications associated with transvenous leads. Leadless pacemakers (LPs), such as the Micra (Medtronic) and Aveir (Abbott) systems, are entirely self-contained units implanted directly into the right ventricle via a minimally invasive percutaneous approach. While both devices eliminate lead-related complications, they have distinct features: Micra operates as a single-use device with no battery replacement option, whereas Aveir is designed for retrievability and battery exchange, potentially extending device longevity. Additionally, Aveir offers atrioventricular synchrony in its dual-chamber version, enhancing physiological pacing. Similarly, subcutaneous and extravascular implantable cardioverter-defibrillators (S-ICDs and EVDs) represent significant progress in sudden cardiac death prevention. The S-ICD (Boston Scientific), which places the defibrillation lead entirely outside the vascular system along the sternum, eliminates the risks of lead-related venous obstruction and bloodstream infections [19]. However, it lacks pacing capabilities beyond post-shock backup pacing. In contrast, extravascular ICDs (Medtronic), position the lead in the substernal space, enabling both defibrillation and pacing, including anti-tachycardia pacing (ATP), while still avoiding intravascular placement. A key future innovation in the field is the modular therapy approach from Boston Scientific, which aims to integrate leadless pacing with S-ICD therapy. Their next-generation leadless pacemaker is designed to communicate wirelessly with the S-ICD, enabling coordinated ATP delivery for tachyarrhythmia termination, thereby expanding the therapeutic capabilities of leadless systems. These advancements collectively enhance patient safety and broaden the spectrum of options for cardiac rhythm management.

### 2.2. Venous Obstruction Syndrome

Venous obstruction affecting vessels where transvenous leads are placed is one of the potential complications of cardiovascular implantable electronic device (CIED) implantation and, according to published studies, occurs in 14–64% of patients with CIEDs [20]. The wide range reported depends on differences in diagnostic methods used to detect the obstruction, criteria to define the obstruction, study population, and time since leads implantation. Transvenous device leads reach the right ventricular cavity via superior arm veins (cephalic, axillary, subclavian) and central veins (innominate and superior vena cava). Usually, patients with venous obstruction are asymptomatic, and this finding often occurs during electrophysiology procedures, such as device upgrading. Clinical signs and symptoms include the development of collateral circulation and superior vena cava syndrome in case of complete vein engagement. Some authors have proposed a temporal classification to reflect the evidence of obstruction in relation to lead implantation [21,22]. The condition is considered acute when it occurs within days to weeks after implantation, subacute when it develops after several months, and late when it manifests from months to years after the procedure. The pathophysiology of lead-related vein obstruction is not well understood. It is assumed that the process involves endothelial injury by leads, activation of inflammatory cascade, procoagulant factors and fibrosis development [23]. Furthermore, conflicts between CIED leads and permanent venous access devices, such as hemodialysis catheters, venous ports, or arteriovenous fistulas, can lead to venous obstruction, dysfunction, and recurrent infections. Chronic venous occlusion and thrombosis may compromise vascular access in patients requiring long-term hemodialysis, highlighting the need for careful vascular planning and monitoring in patients with multiple intravascular devices [24]. Currently, there are no standardized recommendations for managing venous occlusions, including intervention timing and approach. Management depends on center skills such as anticoagulation therapy, local thrombolytic therapy, thrombus aspiration, transvenous or surgical lead extraction, balloon angioplasty and/or venous stenting [25]. In asymptomatic patients, a conservative strategy is the most common. This includes new lead implantation on the contralateral side with consequent lead tunneling or the use of anticoagulant therapy. In case of impossible contralateral side implantation or in the presence of superior vena cava syndrome signs and symptoms (facial plethora, dyspnea and/or cape oedema), lead extraction is a valid option [26,27]. If symptoms persist, in selected cases, experienced centers may perform balloon angioplasty and venous stenting in combination with lead extraction.

### 2.3. Lead Failure and “Crush Syndrome”

The Heart Rhythm Society (HRS) consensus defines lead failure as the malfunction of any lead component, including insulation, conductors, connectors, terminal pins, electrodes, and coils [28]. We can sort different lead failure types:

- Insulation breach: associated with lead exposure. Characterized by electrical parameter alteration such as abrupt drop of impedance (<200 Ohm) and noise generation.
- Fracture lead: associated with electrical parameters changes such as noise, sudden impedance rise (>2000 Ohm) and abrupt pacing threshold increase [29]. Spontaneous lead fracture is a serious complication that may result in the proximal migration of the broken lead segment within the cardiovascular system. This can lead to embolization, vascular obstruction, arrhythmias, or even end-organ damage, necessitating prompt detection and intervention, often through percutaneous retrieval techniques or surgical extraction.

Lead failure clinical presentations can be various and depend on the type of device and patient features: from completely asymptomatic to an inability to deliver appropriate therapy or inappropriate shocks. Cumulative incidence of lead failure at 1 year was 0.6%; at 5 years, 6.5%; and at 10 years, 16.4%. The highest risk of lead failure was found in small-diameter leads [30]. Currently, remote monitoring has become an invaluable tool in early detection of lead-related issues. By continuously tracking electrical parameters, this technology enables healthcare providers to identify potential lead alterations promptly. When anomalies are detected, patients can be quickly called for a specialized electrophysiology ambulatory check, potentially preventing more serious complication. The most frequent cause of lead fracture is the “crush syndrome”, which typically develops through mechanical stress caused by compression between clavicle and fist rib, and repeated movements that gradually compromise the lead’s structural integrity [31]. Possible treatments of lead failure are the relocation of the lead in case of dislocation. In cases of insulation breach or lead fracture, the treatment of choice is to add a new lead and eventually the extraction of the damaged one. In selected cases, reprogramming the setting of the CIED to avoid or to delay lead replacement is sufficient [32]. Preventive measures include avoiding the subclavian access to prevent crush syndrome, preferring axillary venous or cephalic access [33,34].

### 2.4. Lead Dislocations

#### 2.4.1. Right Heart

Lead dislocations in right chambers are related to a change in lead tip position associated usually with electrical parameter changes [35]. The incidence of lead dislodgement ranged from 1% to 2.69% in individual studies. There was a relatively higher lead dislodgement rate between atrial and ventricular electrodes [36]. We can divide this into micro-dislocations, in which position change is not visible with imaging techniques, and macro-dislocations which are visible on thoracic X-ray. Risk factors for this condition are older age, female sex, atrial fibrillation and frailty of the patient [37]. According to the time criterion, lead dislocations can be subdivided into early, within 30 days from implantation, and late, beyond 30 days from implantation [38].

#### 2.4.2. Left Heart

Left heart lead placement, particularly in the presence of a patent foramen ovale (PFO) or atrial septal defect (ASD), can lead to systemic embolism, posing significant risks for cerebrovascular events and organ infarction. Misplacement of leads into the left atrium or ventricle can occur inadvertently and requires immediate recognition and correction to prevent thromboembolic complications.

#### 2.4.3. Perforation

Late lead perforation is a rare complication of CIED procedures. It is usually divided into two categories: early, occurring within the month of implantation, and late, occurring beyond the month of implantation [39]. Clinical presentation is highly variable, ranging from asymptomatic patients to sudden cardiac death [40]. Electrical parameters may be within the normal range during device check. For these reasons, diagnosing lead perforation is difficult and may be underestimated. A combination of clinical symptoms, chest X-ray, ECG and device check may lead to the diagnosis or suspicion of perforation, but computed tomography (CT) appears to be the most sensitive diagnostic technique [41]. Risk factors for perforation include older age, use of active fixation lead, thin ventricular wall and corticosteroid use [42]. Currently, there is not a shared consensus for the management of lead perforation. In several cases reported in the literature, the lead was extracted using a transvenous approach with the patient under general anesthesia, guided by trans-esophageal echocardiogram and with surgical backup [43].

#### 2.4.4. Left Bundle Branch Area Pacing Displacement

In recent years, conduction system pacing (CSP) has become a routine procedure in many centers, gaining increasing attention as a more physiological alternative to right ventricular (RV) pacing [44,45]. Among CSP techniques, Left Bundle Branch Area Pacing (LBBAP) offers several advantages over His Bundle Pacing (HBP), particularly in cases of distal blocks that cannot be treated with HBP [46]. Additionally, LBBAP can serve as an alternative to biventricular pacing, overcoming the limitations associated with left ventricular (LV) epicardial pacing [47]. While LBBAP has proven beneficial, it is not without risks. Procedure-related complications include interventricular septum perforation, lead dislodgement, septal injury, and lead fracture. The MELOS study reported acute and late complications in 11.7% of cases [48]. The most critical phase of the implantation is the penetration of the septum by the lead. During this step, the lead can become entangled, potentially causing entrapment, drilling, screwdriver-like injury, dislodgement, or partial or over perforation [49]. In the MELOS study, complications associated with the transseptal route were observed in 8.3% of patients (209/2533), including delayed septal perforation and coronary artery damage or spasm. Septal perforation can occur in 0–14.1% of patients [50]. One useful indicator of perforation is unipolar myocardial current of injury (COI). A COI ≤ 2.3 mV (mean 0.9 ± 0.6 mV) indicates perforation, while good positioning typically shows a COI around 9 mV. Other signs include a sudden decrease in pacing impedance (below 450 Ω) or a drop in impedance greater than 200 Ω. Acute perforation is usually asymptomatic and can be resolved by repositioning the lead, as dislodgement due to the drilling mechanism can occur. No long-term sequelae have been reported after lead repositioning in observational registries. Late septal perforation occurs at a rate of 0.1–0.3%, and in these cases, repositioning is required, along with the initiation of oral anticoagulation to prevent thromboembolic complications [51]. The rate of lead macro-dislodgement in LBBAP is similar to that seen in standard RV pacing. However, lead micro-dislodgement, which can lead to loss of left bundle capture, is often underestimated. In the MELOS registry, 4% of patients experienced loss of terminal R-wave in V1, and 0.7% (17/2533) had a clinically significant increase in LBBAP pacing threshold, typically detected 7.1 ± 5.0 months after implantation. Mechanical trauma to coronary vessels is also a potential complication, though acute coronary events due to direct damage to septal perforators or coronary artery spasm are rare. These are typically managed conservatively without lasting sequelae. Myocardial infarction, presenting with acute chest pain and ST-segment elevation, has been reported in only a few cases [52,53], and fistula formation involving septal perforators or a coronary venous system and the right ventricle has been described, but it is rare and generally does not lead to clinical consequences. It is important to note that the complication rate associated with the transseptal approach decreases as the operator gains experience. Operator expertise plays a crucial role in successfully penetrating the interventricular septum and minimizing complications [54].

### 2.5. Lead-Dependent Tricuspid Valve Dysfunction

Lead-dependent tricuspid valve dysfunction (LDTVD) is an increasingly recognized complication in patients with cardiac implantable electronic devices (CIEDs), manifesting as tricuspid regurgitation (TR) due to interference of the lead with the tricuspid valve apparatus. The incidence of LDTVD varies widely, with studies reporting a prevalence ranging from 4.4% to 45%, depending on patient selection and follow-up duration [55]. The pathophysiology of LDTVD primarily involves mechanical disruption of the tricuspid valve leaflets, leading to impaired coaptation and valvular incompetence. The most common mechanisms include a propping upward or clamping down of the leaflets by the lead, excessive lead looping at the tricuspid annulus, and adherence of the lead to cardiac structures such as the valve leaflets, right atrium, or right ventricle. These alterations contribute to persistent valve opening and progressive TR, which may ultimately lead to right ventricular dilatation and heart failure symptoms. Risk factors for LDTVD have been extensively analyzed, with significant associations identified for female sex, atrial fibrillation, history of valvular heart disease, previous cardiac surgery, pre-existing TR, and right ventricular dilatation. Other contributing factors include the presence of a high-voltage lead, a greater number of leads traversing the tricuspid valve, and specific lead positions that interfere with the tricuspid annulus, leaflets, or chordae tendineae [56]. Echocardiographic findings have demonstrated that excessive lead loops within the tricuspid valve or adhesions between the lead and cardiac structures increase the likelihood of TR progression. Management strategies for LDTVD remain challenging and require individualized approaches based on symptom severity and the extent of TR. Conservative management with diuretics and afterload reduction may be sufficient for patients with mild TR, but those with severe, symptomatic TR often require intervention. Transvenous lead extraction (TLE) has been proposed as a potential therapeutic option, particularly for patients with severe TR and right heart failure. Studies have shown that TLE can lead to significant improvements in TR severity in approximately 35% of cases, particularly when TR is directly caused by lead interference rather than secondary right ventricular dysfunction [57]. However, the success of TLE in reversing TR depends on factors such as lead dwell time, the presence of severe right ventricular remodeling, and the persistence of annular dilatation post-extraction. Despite its potential benefits, TLE carries inherent risks, including tricuspid valve damage, pulmonary embolism, and cardiac perforation, necessitating careful patient selection and procedural expertise. Alternative strategies, such as lead repositioning or implantation of leadless pacing systems, may be considered in select patients to mitigate the risk of recurrent LDTVD. Additionally, novel imaging modalities, including 3D transesophageal echocardiography, are increasingly utilized to assess lead positioning and guide management decisions. In conclusion, LDTVD represents a significant complication of CIED implantation, contributing to progressive TR and adverse cardiovascular outcomes. Identification of high-risk patients, optimization of lead placement during initial implantation, and timely intervention in symptomatic cases are essential to improving clinical prognosis [58]. Future research should focus on refining risk stratification models, evaluating long-term outcomes following TLE, and exploring novel pacing technologies to minimize the incidence of LDTVD.

## 3. Functional Disorders

### 3.1. CIED-Mediated Arrhythmias

Pacemaker-mediated arrhythmias (PMAs) represent a subset of cardiac rhythm disturbances directly or indirectly caused by the functioning of an implanted cardiac pacemaker. Indeed, the interaction of a pacemaker with the heart’s intrinsic electrical activity can occasionally lead to the initiation of different forms of arrhythmias. Correct programming of the device is essential to avoid or manage this type of arrhythmic episode [59,60,61]. Such arrhythmias are often complex, involving feedback loops between the pacemaker and the heart’s electrical system. PMAs can lead to symptoms like palpitations, fatigue, syncope, or, in severe cases, hemodynamic instability. Understanding PMAs involves analyzing the interplay between pacemaker settings, lead positioning, and the patient’s electrophysiological characteristics. In some cases, pharmacological intervention or an electrophysiological study may be required to resolve these arrhythmias.

#### 3.1.1. Endless Loop Tachycardia (ELT)

Endless loop tachycardia is a specific type of pacemaker-mediated reentrant tachycardia.

**Figure 3:**
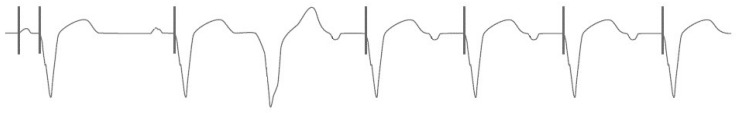
Pacemaker mediated tachycardia.

Clinically, patients may exhibit symptoms such as palpitations, dizziness, shortness of breath or even syncope. In patients with heart failure, symptoms of heart failure may be exacerbated, and, in the worst cases, the condition may lead to hypotension and hemodynamic instability. The mechanism of the tachycardia results from the retrograde conduction of a ventricular beat through the atrioventricular (AV) node (typically through the slow pathway) or even through an accessory pathway. It can be triggered by a premature ventricular beat (PVC) or a paced beat with a long AV interval (Figure 3). Retrograde V-A conduction activates the atrium, given an atrial event is sensed by the pacemaker. This latter one triggers ventricular pacing. The retrograde conduction of the paced ventricular beat perpetuates the arrhythmia. Technically, it can be defined as a reentrant tachycardia where the antegrade limb is the pacemaker, and the retrograde limb is the ventriculoatrial (V-A) retroconduction. The rate of the PMT is the result of the sum of the AV delay and the time of V-A retroconduction. The maximum rate of a PMT is always less or equal to the programmed upper tracking limit [62]. This arrhythmia can be usually prevented by programming the post-ventricular atrial refractory period (PVARP) to be longer than the retrograde V-A conduction interval. However, a long PVARP can limit the upper rate of the pacemaker, potentially causing an electronic atrioventricular 2:1 block when the atrial rate is faster (for example, during exercise). A temporary solution in the case of repetitive ELT may be reprogramming the device in DDI mode impeding the tachycardia. Some manufacturers adopt criteria to define and interrupt PMTs that do not require manual adjustment of PVARP [63].

#### 3.1.2. Repetitive Non-Reentrant Ventriculoatrial Synchrony

A less known arrhythmia that can be triggered by a DDD pacemaker is repetitive non-reentrant ventriculoatrial synchrony (RNRVAS). The clinical presentation is similar to that of PMTs, even though the electrophysiological mechanism is slightly different. This type of tachycardia is also sustained by retrograde V-A conduction. In this case, atrial activity falls within PVARP so that it is not sensed, but it makes the atria refractory to the following atrial paced beat. Ventricular pace is delivered at the end of the programmed AV delay causing a new retrograde VA conduction and the cycle repeats again. RNRVAS is promoted by long AV intervals, long PVARP or a high lower rate limit or any feature, such as rate response, which allows rapid pacing. Moreover, RNRVAS can also trigger automatic mode switching (AMS) because all atrial events are used to calculate the atrial rate [64]. Reducing the PVARP may favor the resolution of the arrhythmia. Rate-response algorithms (DDDR and DDIR modes) may also be responsible for sustaining tachycardia. Decreasing the lower rate limit and/or the rate-response sensor limit may allow the completion of the atrial refractory period, such that a paced atrial event after a retrograde *p* within PVAPR might not result in functional non-capture and prevent the development of the arrhythmia.

#### 3.1.3. The 2:1 Lock-In—Mode Switching Failure During Atrial Flutter

Normally, automatic mode switching algorithms prevent the tracking of supraventricular arrhythmias. This avoids atrial activity during atrial fibrillation or atrial tachycardia from being followed by ventricular pacing at the upper tracking limit. However, a failure of the algorithm may produce a phenomenon called 2:1 lock-in. This behavior is the result of an atrial event which falls into the post-ventricular atrial blanking period (PVAB). Since the upper tracking limit of a pacemaker is usually over 120 bpm, the phenomenon happens in case of atrial flutters. The typical sequence includes a first atrial sensed event, which triggers an AV interval. Subsequently, the second atrial event falls within the PVAB, and it is not sensed at all. The third atrial event falls outside the atrial refractory period, and it is sensed so it triggers an AV interval and so on. Atrial rate appears as half of the real one and below the maximum tracking rate (MTR) failing to trigger AMS. This usually happens in patients with dual chamber pacemakers programmed in DDD mode, spontaneous or radiofrequency-induced AV-block and paroxysmal atrial flutter. In order to avoid this phenomenon, the AV delay should be programmed to be as short as possible as it increases the chance that the atrial events will be sensed out the PVAB [65]. Another option is to decrease the MTR.

#### 3.1.4. Short-Long-Short Sequences Promoting Ventricular Tachyarrhythmias

**Figure 4:**
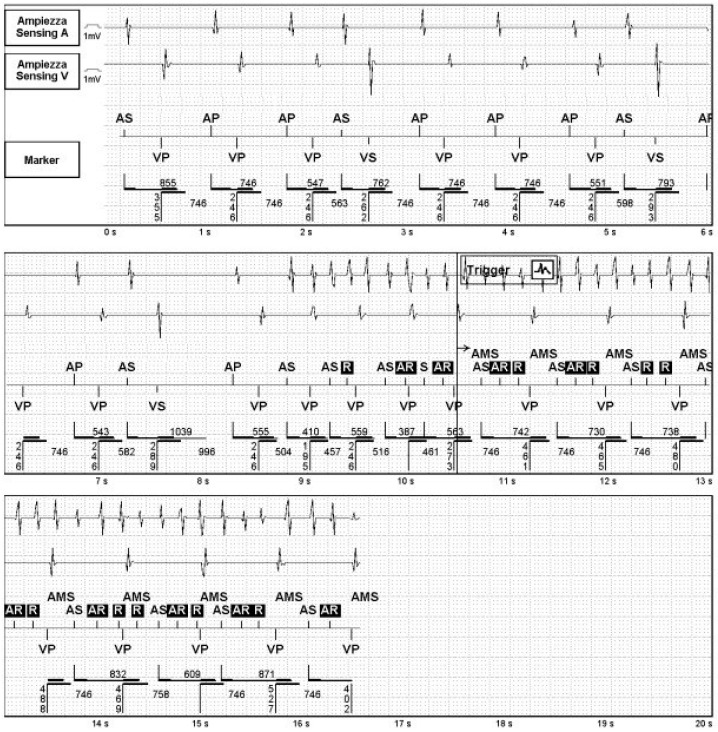
Short-long-short sequence inducing atrial fibrillation.

In patients with CIEDs, short-long-short sequences induced by pacing may contribute to the genesis of tachyarrhythmias (Figure 4).

Many mechanisms, such as a sudden AV block during sinus tachycardia or atrial fibrillation and subsequent backup pacing, AV hysteresis [66], ventricular safety pacing [67], may promote the phenomenon. An analysis of the initiating sequences of the VT/VF episodes in the Painfree RX II and Entrust Trial showed that, in patients with implantable cardioverter-defibrillator (ICD) pacing-facilitated short-long-short sequences accounted for 8% to 15% of all VT/VF and was the only onset sequence in up to 10% of patients with VT/VF [68]. Most patients showed monomorphic scar-related tachycardias, and the short-long-short sequence would be responsible for triggering the reentry phenomenon around the scar. The short-long-short ventricular sequences function as extrastimuli capable of promoting dispersion of ventricular repolarization, slow conduction or unidirectional block favoring reentry mechanisms or triggering polymorphic tachycardias such as torsades de pointes. One of the solutions is to avoid pacing modes that permit transient bradycardia such as lower rate programming, AV hysteresis or algorithms aiming to reduce ventricular pacing.

### 3.2. Defibrillator-Specific Complications

#### 3.2.1. Inappropriate Shocks

In the patient’s medical records, an ICD shock was classified as appropriate if it occurred in response to a ventricular tachyarrhythmia that met the criteria established by the device programming. Conversely, any shock failing to meet these criteria was considered inappropriate. Inappropriate shocks may arise from atrial arrhythmias with rapid ventricular conduction, abnormal sensing (e.g., T-wave oversensing), or issues such as lead artifacts or ICD malfunctions. Receiving an ICD shock is highly distressing for patients and can often be a traumatic experience. Studies have shown that such shocks, aside from reducing quality of life, are linked to increased mortality, particularly in cases where atrial fibrillation (AF) triggers inappropriate shocks [69]. Similarly, the MADIT-II trial showed that the occurrence of an inappropriate shock is associated with a doubled risk of mortality for all causes, likely due to potential direct mechanical, arrhythmic, or hemodynamic adverse effects of the shocks themselves, as well as baseline characteristics such as the presence of AF [70]. Kleemann et al. compared the prognostic impact of inappropriate shocks related to atrial fibrillation versus those related to lead failure, highlighting that multiple ICD shocks triggered by AF are associated with worse prognosis, whereas a single shock due to AF or shocks resulting from lead failure are not [69]. The incidence of inappropriate shocks reported in MADIT-II and similar studies [71,72,73] ranged between 10% and 15%. However, the more recent CARAT observational study reported a significantly lower incidence of 1.6%, focusing on all approved and marketed ICD systems [74]. Another study by Oosterwerf et al. documented an 8.7% rate of inappropriate shocks in patients with subcutaneous ICDs (S-ICDs), with a higher frequency observed in individuals with nonischemic cardiomyopathy compared to those with ischemic cardiomyopathy [75]. Predictors of inappropriate shocks include age below 70 years, nonischemic heart disease, elevated resting heart rate, and the occurrence of prior appropriate shocks [76]. The primary parameter initiating ICD therapy is the patient’s heart rate exceeding a set threshold for a predefined duration. Randomized prospective studies have demonstrated that programming therapy zones to deliver shocks only at higher rates or after a longer detection period for ventricular arrhythmias can significantly reduce shocks due to supraventricular tachycardia (SVT) or non-sustained ventricular tachycardia (VT). The PREPARE study first demonstrated the benefits of extended detection durations (30–40 beats) by comparing outcomes to a historical ICD cohort with conventional detection settings. This approach reduced inappropriate shocks for SVTs and preventable shocks for VT [77]. Similarly, MADIT-II reported a decrease in inappropriate shocks alongside reductions in all-cause mortality.

**Figure 5:**
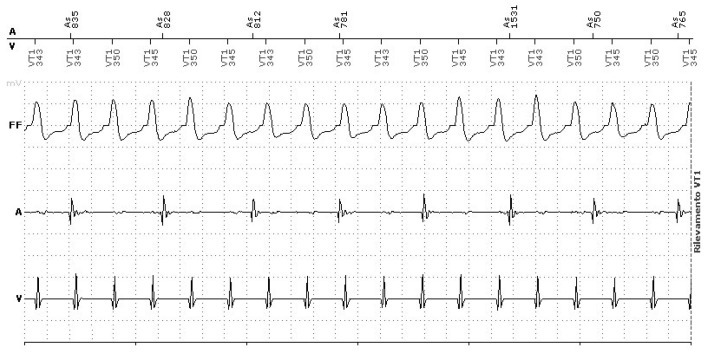
Ventricular tachycardia EGM’S. AV dissociation.

A key consideration in ICD programming is the ability to distinguish VT from other rapid rhythms. Therapy can be withheld if specific criteria, such as arrhythmia stability, R-wave morphology, or tachycardia onset, are satisfied. Dual-chamber ICD systems enhance this process by evaluating atrial and ventricular events and their interrelationship (Figure 5). However, more aggressive discriminator settings may increase the risk of failing to deliver appropriate therapy when required [78]. Beyond optimizing programming, pharmacological treatments and interventions like AV node ablation or pulmonary vein isolation may serve as complementary strategies to lower the incidence of inappropriate shocks.

#### 3.2.2. Phantom Shocks

Phantom shocks (PS) refer to the perception of receiving a shock without any recorded defibrillation upon interrogation of the ICD device [79]. This phenomenon was first documented in 1992. The reported incidence among ICD patients varies widely, ranging from 2% to 25.5% [80]. The pathophysiology underlying PS remains poorly understood. Several factors have been implicated in their development. Varghese et al. identified a strong association between a history of appropriate ICD shocks and the occurrence of phantom shocks [81]. Similarly, Jacob et al. reported that patients experiencing PS were more likely to have pre-existing depression and anxiety compared to those without [82]. Patients with ICDs often face profound lifestyle changes, including altered body image, occupational restrictions, and persistent fear of future shocks or life-threatening events. Studies indicate a general decline in perceived quality of life among ICD recipients, particularly in areas such as physical activity and family dynamics [83]. Both appropriate and phantom shocks, which are indistinguishable from the patient’s perspective, frequently evoke alarm, frustration, and confusion. This leaves patients grappling with the uncertainty and emotional burden of a distressing and unpredictable experience [84]. The psychological consequences can be significant, with some individuals developing severe anxiety or depression. Currently, there is no specific treatment protocol for managing phantom shocks. However, several studies have explored potential preventive and therapeutic strategies. Berg et al. investigated a combined rehabilitation approach that incorporated physical training and psychoeducational components to enhance overall quality of life in ICD patients and potentially prevent PS. While this intervention improved mental health outcomes, it did not effectively reduce the incidence of phantom shocks [85]. In another study, Lewin et al. developed the “ICD Plan,” which evaluated the impact of cognitive-behavioral therapy (CBT) compared to standard care in mitigating the psychological impact of living with an ICD. Although participants in the CBT group reported fewer physical limitations and an improved quality of life, the intervention did not produce a statistically significant effect on the frequency of shocks or phantom shock symptoms [86].

#### 3.2.3. Arrhythmic Storms

An electrical storm (ES) is defined as the occurrence of three or more distinct episodes of ventricular tachycardia (VT) or ventricular fibrillation (VF) within a 24-h period, with each episode separated by at least five minutes [87]. This condition represents a state of profound electrical instability, arising from a rare combination of factors, and is associated with hemodynamic compromise, potential mortality, and, in patients with implantable cardioverter defibrillators (ICDs), repeated shocks. Evidence indicates that ES serves as a negative prognostic indicator. It correlates with increased mortality rates in both primary and secondary prevention populations, alongside higher hospitalization rates, and significantly diminishes patients’ quality of life. ES has been identified as an independent risk factor for mortality, unrelated to left ventricular ejection fraction or other prognostic variables. The risk of death is particularly elevated during the first three months following an ES, gradually decreasing thereafter [88]. However, it remains uncertain whether ES directly contributes to higher mortality or simply reflects the progression of advanced cardiac or systemic disease. While no definitive triggers for ES have been established, clinical studies have linked its occurrence to factors such as severely impaired left ventricular function, chronic renal dysfunction, ischemia, infections, electrolyte imbalances (e.g., hypokalemia or hyperkalemia), and advanced age [89,90]. As a medical emergency, ES requires prompt and aggressive management of any identifiable triggers. For patients experiencing frequent shocks, sedation may be employed to mitigate psychological distress [91]. The first-line treatment for ES is typically pharmacological, involving beta-blockers, amiodarone, and occasionally other antiarrhythmic agents. Early consideration of catheter ablation is recommended, particularly in cases of ischemic cardiomyopathy. Recent findings from the STAR study, a large multicenter investigation, support the safety and efficacy of percutaneous left stellate ganglion blockade for managing refractory ES [92,93,94]. Conversely, aggressive ICD programming—characterized by lower VF detection thresholds, reduced detection times, and the exclusion of antitachycardia pacing (ATP) during capacitor charging—has been associated with an increased incidence of ES. This may be due to the higher number of short, self-terminating arrhythmic episodes detected and treated by the device, raising the likelihood of ES and its associated risks, including death and heart failure.

ATP is widely recognized as an effective technique for terminating certain arrhythmias, particularly slow monomorphic VT caused by reentry circuits. The principle involves delivering pacing stimuli at a rate faster than the VT, exploiting the excitable gap within the reentrant circuit. This interrupts the tachycardia by creating new activation wavefronts that collide with the existing arrhythmic wavefront, effectively terminating it.

In ICD patients, particularly those with prior myocardial infarction, ATP may either terminate VT or, in some cases, alter it into another VT or even VF. Adduci et al. observed that ATP successfully terminated 69% of monomorphic VT episodes in their cohort of 39 cases. However, in 18% of interventions, ATP converted the initial VT into a new VT. Additionally, ATP was applied inappropriately in 27 out of 42 detected episodes, inducing two de novo VTs (7%) [95]. The mechanisms underlying ATP-induced VTs remain poorly understood, and it is unclear if these arrhythmias carry the same prognostic weight as spontaneous VTs. Sharaf-Dabbagh et al. found that ATP-induced VTs differ from spontaneous VTs in several ways: they tend to be faster, less confined to the endocardium, and less easily inducible. These differences suggest that ATP-induced VTs may arise from predominantly functional circuits, which limits the utility of pace-mapping to identify ablation targets [96]. Their study concluded that ATP-induced VTs are not directly causative of VT recurrence but rather serve as markers of recurrence. Ablation of spontaneous VTs effectively prevented recurrence of ATP-induced VTs, further supporting the notion that these arrhythmias are distinct from their spontaneous counterparts.

### 3.3. Resynchronization Therapy Specific Complication

#### 3.3.1. Arrhythmogenic Potential from Coronary Sinus Pacing

Cardiac resynchronization therapy (CRT) has emerged as a valuable adjunctive treatment to optimize pharmacological management in patients with heart failure and bundle branch block [97,98,99]. Long-term studies have demonstrated its benefits, including reverse ventricular remodeling, enhanced exercise capacity, improved functional class, better quality of life, and reduced hospitalizations [100,101]. However, the relationship between CRT and sudden cardiac death (SCD) or the risk of ventricular arrhythmias (VA) induction remains a topic of ongoing debate.

On the one hand, the extent of reverse remodeling achieved with CRT is inversely associated with the risk of future VA events, as observed in the MADIT-CRT trial [102]. On the other hand, CRT, particularly epicardial left ventricular (LV) pacing via the coronary sinus, has been implicated in the promotion of ventricular arrhythmias. In large-scale studies such as COMPANION [103] and CARE-HF, CRT was associated with reductions in all-cause mortality. However, a higher proportion of deaths classified as sudden occurred in CRT recipients compared to those receiving optimal pharmacological therapy alone. A prospective Italian registry of 649 CRT-D patients reported 169 episodes of electrical storm (ES) in 45 patients (7.1%) during a mean follow-up of 19 ± 11 months [104]. Of these, eight patients (1.3%) experienced ES within the first 3 weeks post-implantation, with CRT interrupted in two cases due to ES directly linked to LV epicardial stimulation. The median time from implantation to the first ES was 6 months (interquartile range 3–15 months). Case reports have highlighted the proarrhythmic effects of CRT, often characterized by monomorphic reentry VT triggered by LV pacing alone, which could be mitigated by activation of biventricular pacing [105,106,107]. LV epicardial pacing alters the normal sequence of ventricular activation, causing delayed endocardial depolarization and repolarization. This results in prolonged action potential duration, QT interval extension, and increased transmural dispersion of repolarization, all of which enhance arrhythmogenic risk [108]. To address this, certain manufacturers have developed LV lead sensing features with T-wave protection to avoid pacing during the vulnerable phase of the T wave [109]. The arrhythmogenic potential of CRT is influenced by various factors, including the underlying myocardial substrate, autonomic tone modulation, and triggers such as ischemia or premature ventricular contractions (PVCs). Surface ECG markers such as QTc and Tp-e intervals, which reflect transmural dispersion of repolarization, may serve as predictors of major arrhythmic events [110]. A study by Kawamura et al. [111] found that increases in LV pacing thresholds were associated with higher VA frequency. Patients requiring higher pacing thresholds due to lead dislodgment or loss of capture exhibited prolonged trans-ventricular conduction times and QTc interval extension (from 539 ± 45 ms at thresholds < 0.5 V to 559 ± 46 ms at thresholds > 5 V). Furthermore, patients who did not respond to CRT, in terms of NYHA functional class improvement or LVEF increase, had a higher incidence of VA and ES compared to responders. Subgroup analyses from the COMPANION trial indicated a trend toward higher SCD rates in patients with CRT-pacemakers (CRT-P) (7.8%) compared to those receiving optimal medical therapy (5.8%) over 16 months. However, studies like REVERSE [112] and RAFT [113] which included patients with mild to moderate heart failure, reported no significant difference in VA rates between CRT-D and ICD-only groups. This neutral effect may reflect a balance between increased arrhythmic risk in non-responders and reduced risk in responders [114]. LV reverse remodeling, rather than symptomatic heart failure improvement, appears to be a key predictor of reduced VA risk. Changes in LV geometry are thought to influence myocardial electrophysiological properties, with Itoh et al. [115] demonstrating a progressive reduction in repolarization markers (e.g., T peak to end) alongside decreases in LV end-systolic volume (LVESV) and VA rates over time. In summary, while CRT exerts antiarrhythmic effects through LV reverse remodeling, it may also promote arrhythmias in the absence of remodeling. Optimizing patient selection, lead positioning, and biventricular pacing are essential to enhancing CRT response and minimizing proarrhythmic risks [116].

#### 3.3.2. Phrenic Nerve Stimulation in LV Epicardial Pacing

One significant limitation of cardiac resynchronization therapy (CRT) is the potential for phrenic nerve stimulation (PNS), caused by electrical impulses delivered by the left ventricular (LV) lead. The therapeutic benefit of CRT, including improvements in LV volume and function, depends heavily on lead positioning at the site of delayed LV activation, which is often near the lateral or posterior heart walls, close to the phrenic nerve. PNS, reported in 2% to 37% of patients across various trials, is a clinically relevant complication that can compromise the effectiveness of CRT [117,118,119]. PNS may manifest as continuous or intermittent episodes, causing symptoms such as dyspnea, muscle twitching, hiccups, or general discomfort. This issue is not always evident during implantation, as PNS is often position-dependent and may emerge later due to changes in patient posture or lead stability. Anatomically, the phrenic nerve is closest to the coronary veins along the lateral and inferior heart walls, with an average distance of 3.4 ± 4.3 mm from the left marginal vein, compared to greater distances from the coronary sinus (7.3 ± 4.7 mm) and anterior interventricular vein (6.3 ± 3.8 mm). Apical lead positions also pose a higher risk of PNS compared to basal positions [120]. Early CRT systems offered limited pacing configurations, such as pacing from a distal electrode (LV tip to can or LV tip to RV ring). Bipolar leads later introduced four pacing options, but it is the advent of quadripolar leads that has significantly enhanced flexibility. Quadripolar leads allow for multiple pacing configurations, reducing the likelihood of PNS and high pacing thresholds without requiring lead relocation. “Electronic repositioning”, achieved by reprogramming the pacing vector of the LV lead, has proven effective in resolving PNS. In contrast, older systems often necessitated physical lead repositioning [121]. Behar et al. [122] found that during follow-up, PNS occurred in 16.0% of patients with quadripolar leads compared to 11.6% with bipolar leads (*p* = 0.08). In the quadripolar group, reprogramming successfully eliminated all cases of PNS, whereas only 60% of PNS cases in the bipolar group were resolved through reprogramming, with the remaining 40% requiring lead repositioning. The EffaceQ study [123] demonstrated that quadripolar LV leads provide superior flexibility, offering a higher number of viable pacing configurations and alternatives for electronic vector reprogramming. This approach reduces the need for invasive interventions when managing high pacing thresholds or PNS. Furthermore, data from an Italian multicenter study indicated that quadripolar active fixation LV leads were associated with fewer complications and increased capacity for electronic repositioning compared to bipolar leads, optimizing CRT delivery and minimizing the impact of PNS [124,125].

### 3.4. Hemodynamic Issues

#### 3.4.1. Pacemaker Syndrome

Pacemaker syndrome was first described in 1969 by Mitsui et al. as a collection of symptoms related to the intolerance of poor coordination between atrial and ventricular contractions, resulting in a reduction in cardiac output during pacing rhythms. There is no global consensus on a standardized definition for pacemaker syndrome. In the PACE (Pacemaker Selection in the Elderly) study, it was defined as symptoms severe enough to prompt reprogramming to dual-chamber pacing [126]. In contrast, the MOST (Mode Selection Trial) defined pacemaker syndrome based on two possible criteria: (1) new or worsened symptoms related to ventricular conduction during ventricular pacing, or (2) symptoms associated with a >20 mmHg drop in systolic blood pressure during VVIR pacing compared to atrial pacing or sinus rhythm [127]. The incidence of pacemaker syndrome varies widely, from 5% to 80%, and it is most commonly associated with the VVI pacing mode, which is more prone to causing atrioventricular dyssynchrony [128]. Symptoms of pacemaker syndrome include dyspnea, orthopnea, fatigue, dizziness, confusion, reduced exercise capacity, and syncope. These symptoms result from a reduction in cardiac output, leading to hypotension. The back pressure in the venous circulation causes neck vein distension, cannon waves in jugular vein pressure tracing, and lower extremity edema. Although pacemaker syndrome is more frequently associated with VVI pacing, it can occur with any pacing mode that creates an adverse hemodynamic profile, especially when atrial contraction occurs against a closed atrioventricular valve or too close to ventricular contraction. This results in back-pressure in the venous circulation, loss of atrial contribution to ventricular output, and a reduction in cardiac output. These changes lead to increased filling pressures and reflex vasodilation, which reduces peripheral resistance. Levels of atrial natriuretic peptide (ANP), a potent vasodilator, are elevated in patients with pacemaker syndrome due to atrial distension and increased arterial and pulmonary pressure, contributing to a drop in peripheral resistance [129]. There are no specific patient characteristics that predict the development of pacemaker syndrome. Management of patients experiencing pacemaker syndrome with VVI pacing involves reprogramming or upgrading to DDD pacing.

#### 3.4.2. Left Ventricular Dysfunction from Right Ventricular Pacing

It is well-established that right ventricular (RV) pacing can negatively impact myocardial function, especially in patients with pre-existing left ventricular (LV) dysfunction. The adverse effects of RV pacing include remodeling and deterioration of both systolic and diastolic function due to the electrical and mechanical dyssynchrony induced by non-physiological pacing. This dyssynchrony results from an altered pattern of ventricular activation, which is strongly influenced by the pacing site, with the RV apex having the most detrimental effect on biventricular function [130]. Chronic RV apical pacing leads to myocardial remodeling, inducing ventricular dilation, asymmetric LV hypertrophy, thinning, altered perfusion distribution, increased myocardial catecholamine concentrations, and abnormal histological changes, such as myofiber disarray. These changes contribute to impaired LV function. Large trials involving pacemakers [131,132,133,134] and implantable cardioverter defibrillators [135,136] have further demonstrated the relationship between RV pacing and adverse cardiovascular outcomes. Specifically, RV pacing results in regional differences in myocardial perfusion and oxygen consumption, reducing myocardial mechanical efficiency.

In a study assessing regional myocardial blood flow (MBF) and global LV ejection fraction (LVEF) in patients with sick sinus syndrome (SSS) randomized to either single-chamber atrial (AAI) or dual-chamber (DDD) pacing, it was shown that during temporary AAI pacing, MBF was significantly higher than during DDD pacing in the inferior (*p* = 0.001) and septal (*p* = 0.004) regions, as well as globally (0.61 ± 0.15 vs. 0.53 ± 0.13 mL·g^−1^·min^−1^, *p* = 0.005). Moreover, in the DDD group, LVEF decreased from pacemaker implantation to the time of MBF measurements (0.61 ± 0.09 vs. 0.56 ± 0.07, *p* = 0.013), while LVEF during temporary AAI pacing was not different from the LVEF at pacemaker implantation [137]. Clinical evidence of the deleterious effects of RV pacing was demonstrated in the Dual Chamber and VVI Implantable Defibrillator (DAVID) trial. This trial tested the hypothesis that dual-chamber pacing for rate support would be more efficacious than backup pacing in patients with impaired ventricular systolic function receiving a dual-chamber implantable cardioverter-defibrillator (ICD). The study was prematurely halted due to increased mortality and hospitalization rates in patients treated with dual-chamber pacing [138]. Liu et al. studied the acute effects of RV apical pacing on LV function using real-time 3D echocardiography in 35 patients with sick sinus syndrome. During RV apical pacing, patients showed a decrease in LV end-diastolic volume (from 79 ± 22 to 76 ± 20 mL, *p* = 0.07) and a reduction in LV ejection fraction (from 57 ± 8% to 54 ± 8%, *p* = 0.01) [139]. Furthermore, Lieberman et al. found that RV apical pacing in patients with preserved LV ejection fraction induced a moderate decrease in LVEF (from 51 ± 12% to 48 ± 14%, *p* = NS), while LV dimensions remained unchanged [140]. Several mechanisms contribute to the decrease in LV end-diastolic volume and LV ejection fraction during RV apical pacing. Early activation of the RV may impede LV filling, which depends on the shared interventricular septum. Additionally, the loss of normal atrioventricular conduction may decrease left atrial contribution to diastolic filling, leading to reduced LV preload and, consequently, decreased cardiac output (according to the Frank–Starling law) [141,142]. Liu et al. also demonstrated an acute increase in LV systolic dyssynchrony, assessed by real-time 3D echocardiography, during RV apical pacing in a group of patients with sick sinus syndrome (from 5.3 ± 2.1% to 7.0 ± 2.5%). In another study, the synchronicity of the LV was assessed using 2D speckle-tracking radial strain imaging, which measured the time difference between the earliest and latest activated segments. The results showed a significant increase in the time difference during RV-paced rhythms, reflecting more dyssynchronous LV contraction. Notably, nine patients (36%) exhibited significant LV dyssynchrony (130 ms between the earliest and latest activated segments) during RV pacing [143]. This dyssynchrony arises from the spreading of the depolarization impulse through the slower-conducting myocardium rather than the His-Purkinje system, leading to heterogeneous electric and mechanical activation of the LV. The study also demonstrated changes in LV systolic mechanics, including a decrease in LV longitudinal shortening (18.3 ± 3.5 vs. 11.8 ± 3.6, *p* < 0.001) and LV twist (12.4 ± 3.7° vs. 9.7 ± 2.6°, *p* = 0.001) when comparing sinus rhythm and RV apical pacing. Sorger et al. observed a dramatic decrease in LV twist during RV apical pacing compared to atrial pacing (6.1 ± 1.7° vs. 11.1 ± 3.5°, *p* = 0.001), measured by magnetic resonance [144]. The detrimental effects of RV pacing appear to be related to the percentage of time spent in paced rhythm. In the MOST trial, ventricular pacing in the VVIR mode for more than 80% of the time was associated with increased heart failure risk. Similarly, RV pacing in the DDDR mode for more than 40% increased the risk of heart failure by 2.6-fold. Chronic RV pacing was also linked to an increased risk of atrial fibrillation (AF). The DAVID trial found that patients with less than 40% DDDR RV pacing had similar or better outcomes compared to the VVI backup group (mean RV pacing < 4%), whereas those with more than 40% RV pacing had worse outcomes. Preserving physiological ventricular activation during permanent cardiac pacing is crucial for optimizing hemodynamic function. There is a growing trend to minimize RV pacing as much as possible. Given the risks associated with RV pacing, especially in patients with pre-existing heart failure, various strategies have been developed to minimize RV pacing. These include using backup ventricular pacing (VVI or VVIR modes), lengthening AV intervals during dual-chamber pacing, programming pacing rates lower than sinus rhythm, or incorporating rate-adaptive AV delays to allow longer AV intervals at lower heart rates and more appropriate AV intervals at higher rates. Additionally, algorithms for minimizing ventricular pacing are now available, which provide AAI(R) pacing with ventricular monitoring and backup support via DDD(R) pacing if a high-degree AV block develops. The SAVE PACe study demonstrated that these algorithms reduced the risk of AF without increasing heart failure events or mortality in patients with sinus node disease [145]. Another approach to minimize RV pacing is the use of AV search hysteresis during DDD pacing, which prolongs the AV delay after a predetermined number of atrial beats to check for spontaneous AV conduction. The INTRINSIC RV trial showed that the use of AV search hysteresis resulted in lower rates of adverse clinical events when RV pacing was between 10 and 19% [146]. For patients with pre-existing heart failure and no preserved AV conduction, where reducing the percentage of RV pacing is not feasible, conduction system pacing (His bundle pacing or LBBAP) is an emerging strategy to reduce RV pacing-related cardiomyopathy.

## 4. Future Directions

The evolution of CIEDs continues to address the challenges of device-related complications while advancing the capabilities of these life-saving technologies. Future research should focus on the development of innovative materials and designs to minimize infection risks, reduce mechanical failures, and enhance device durability. Remote monitoring systems with advanced artificial intelligence (AI) algorithms hold the potential to provide real-time data analysis, enabling early detection and prevention of complications. Furthermore, the integration of leadless and wireless technologies may significantly lower the risks associated with traditional lead-based systems. A key area for exploration is the optimization of personalized device programming, tailored to the unique anatomical, physiological, and lifestyle needs of individual patients. This patient-specific approach, supported by big data analytics, could improve therapeutic outcomes while reducing adverse events. Finally, multidisciplinary collaboration between engineers, clinicians, and researchers is essential to drive innovation in device design, procedural techniques, and patient care strategies.

## 5. Conclusions

The widespread adoption of CIEDs has undeniably transformed the management of cardiac arrhythmias, significantly improving the quality of life and survival rates for millions of patients worldwide. However, the emergence of device-related complications underscores the importance of comprehensive strategies for prevention, early detection, and management. Understanding the mechanisms underlying these complications and leveraging technological advancements can pave the way for safer and more effective therapies. The cornerstone of CIED-related complication prevention lies in meticulous implantation technique. Long-term outcomes are significantly influenced by decisions and technical precision during the initial implantation procedure. Critical factors include optimal pocket creation, careful vessel entry technique, appropriate lead positioning, and proper lead fixation. Studies have demonstrated that implantation-related imperfections, such as inappropriate pocket depth, excessive lead slack, or suboptimal lead positioning, can manifest as complications months or years after the procedure. For instance, superficial pocket creation may lead to eventual erosion, while excessive lead length in the pocket may result in lead migration or twiddling syndrome. Furthermore, the relationship between implanter experience and complication rates has been well documented, with higher-volume operators generally achieving better outcomes. This underscores the paramount importance of structured training programs for new practitioners, incorporating both technical skills and decision-making capabilities. While technological advancements in device and lead design continue to evolve, the fundamental aspects of proper implantation technique remain crucial for optimal long-term outcomes. Healthcare providers must remain vigilant in balancing the benefits and risks associated with CIED therapy. Through continuous education, research, and innovation, the medical community can ensure that these devices continue to provide maximum benefits while minimizing complications, ultimately improving patient outcomes and advancing the field of cardiac electrophysiology.

## Data Availability

Data sharing is not applicable.
